# The Diagnostic Interview for Sexual Dysfunctions in Women for DSM‐5 and ICD‐11: Development and initial validation using a vignette‐based approach

**DOI:** 10.1002/mpr.2004

**Published:** 2023-12-28

**Authors:** Rebekka Schwesig, Julia Velten, Jürgen Hoyer

**Affiliations:** ^1^ Institute for Clinical Psychology and Psychotherapy Technische Universität Dresden Dresden Germany; ^2^ Mental Health Research and Treatment Center Faculty of Psychology Ruhr University Bochum Bochum Germany

**Keywords:** DSM‐5, ICD‐11, psychometric properties, sexual dysfunctions, structured interview

## Abstract

**Objectives:**

The aim of this study was to evaluate the psychometric properties of the newly developed *Diagnostic Interview for Sexual Dysfunctions* *in* *Women* (DISEX‐F), which covers diagnostic criteria of DSM‐5 and ICD‐11.

**Methods:**

Thirty‐two actresses portrayed 32 cases of female sexual dysfunctions (= standardized patients). To calculate inter‐rater reliability, each standardized patient was interviewed independently by two trained diagnosticians using the DISEX‐F. Interviews were videotaped, and each videotape was evaluated by two other independent diagnosticians. Sensitivity and specificity were calculated by comparing the assigned diagnoses to the target diagnoses pre‐determined in the case vignettes. As a side criterion, the acceptance of the DISEX‐F among diagnosticians was assessed.

**Results:**

Specificity was found to be generally clinically satisfying (DSM‐5: 0.90–0.99; ICD‐11: 0.95–0.99), while sensitivity (DSM‐5: 0.40–0.92; ICD‐11: 0.71–0.96) and inter‐rater reliability (DSM‐5: Cohen's kappa = 0.44–1; ICD‐11: Cohen's kappa = 0.75–0.94) greatly varied between classification systems and disorders. Imprecise acting and false differential diagnostic decisions were identified as major sources of mismatch. The acceptance of the DISEX‐F was high.

**Conclusion:**

Results encourage usage of the DISEX‐F for ICD‐11 diagnoses. Mixed results were found for DSM‐5 diagnoses, which can partly be explained by shortcomings in DSM‐5 criteria.

## 1 INTRODUCTION

An accurate diagnosis is fundamental for adequate and effective psychotherapeutic or medical treatment. Toward this goal, the use of structured interviews is a long‐established standard for diagnosing mental disorders (e.g., Rettew et al., 2009). They promise a more objective, reliable, and valid determination of the diagnosis compared to unstandardized clinical judgments and routine anamneses (Bruchmueller et al., 2011). While classification systems such as the *International Statistical Classification of Diseases* (ICD) of the World Health Organization (WHO) or the *Diagnostic and Statistical Manual of Mental Disorders* (DSM) of the American Psychiatric Association (APA) become increasingly complex, structured interviews facilitate the diagnostic process, minimize typical assessment errors and cognitive biases (e.g., confirmation bias), and aim to guarantee comparability and replicability of research results across studies (Rettew et al., 2009).

### 1.1 Lack of high‐quality instruments for diagnosing sexual dysfunctions

Well‐validated structured interviews for mental disorders such as the *Structured Clinical Interview for DSM‐V* (SCID‐5; First et al., 2016) or the *Composite International Diagnostic Interview* (CIDI; World Health Organization, 1997) are widely used for the diagnosis of mental disorders in clinical practice and serve as established standards in research (see e.g., Jiang et al., 2021; Maske et al., 2015; Osório et al., 2019; Shabani et al., 2021). However, none of these instruments include the criteria for sexual dysfunctions leaving the respective research field without a valid, gold‐standard diagnostic instrument until today. Given the lack of such an interview, self‐report measures such as the *Female Sexual Function Index* (FSFI; Rosen et al., 2000) or the *International Index of Erectile Function* (IIEF; Rosen et al., 1997) are typically used in studies to measure sexual dysfunctions (see also Hatzichristou et al., 2016; Meston et al., 2020). Despite their easy and economic use, they have been criticized for major theoretical, statistical, and psychometric problems, for example, biased results for sexual inactive samples toward dysfunction and lack of validity for the measurement of sexual function beyond sexual arousal (see also Forbes et al., 2014; Meyer‐Bahlburg & Dolezal, 2007). Further, they were developed prior to current classification of sexual dysfunctions and, therefore, do not adequately query current diagnostic criteria for sexual dysfunctions, such as personal distress and symptom duration. To our knowledge, the *Women's Sexual Interest Diagnostic Interview* (WSID; Derogatis et al., 2008) is the only (at least partially) validated diagnostic interview for sexual dysfunctions so far. However, it is limited to the assessment of the Hypoactive Sexual Desire Disorder, is based on diagnostic criteria of DSM‐IV (APA, 1994), and has not been adapted to the diagnostic changes established with the revision of the DSM in 2013.

### 1.2 Effects on clinical research and care

Due to the lack of a gold‐standard diagnostic instrument, there is a high variability across studies on how sexual dysfunctions are diagnosed reducing the validity and comparability of clinical research (McCabe et al., 2016). Different authors have demanded to reduce this problem by using well‐validated, standardized instruments (e.g., McCabe et al., 2016; Simons & Carey, 2001). In clinical care, many practitioners (e.g., psychotherapists and physicians) feel insecure and ill‐equipped to diagnose and treat sexual dysfunctions (Levkovich et al., 2021; Zannoni et al., 2021). A valid, reliable, and easily applicable structured diagnostic instrument for sexual dysfunctions would help to reduce uncertainties in clinical care and enhance diagnostic validity in clinical research. The present paper reports the first data on a new clinical instrument developed to close the above‐mentioned gap, the *Diagnostic Interview for Sexual Dysfunctions* (DISEX, Schwesig, Velten, & Hoyer, 2022).

### 1.3 Divergent diagnostic guidelines in the DSM‐5 and ICD‐11

A key objective in the development process of the DISEX was to make the new instrument applicable for clinical research and practical treatment decisions: Non‐specialized clinicians and researchers should be able to administer the DISEX correctly and assign reliable diagnoses without much additional training. Furthermore, while most health practitioners in member countries of the WHO use the ICD to assign diagnosis (Jakob, 2017), many professionals in the United States and especially in international clinical research use the DSM for assigning diagnoses (APA, 2009, 2013). Hence, diagnostic criteria from both current classification systems, the DSM‐5 (APA, 2013, 2022) as well as the ICD‐11 (WHO, 2019) needed to be included in the DISEX to ensure its usefulness across contexts and countries. Unfortunately, diagnostic guidelines for sexual dysfunctions in the ICD‐11 and DSM‐5 differ fundamentally (Schwesig, Briken, et al., 2022): While the ICD‐11 follows a symptom‐driven, multifactorial approach for which the etiology of a sexual problem is irrelevant for the diagnostic decision (exception: Sexual pain‐penetration disorder), non‐mental causes such as medication, injury or disease, as well as non‐sexual mental disorders, significant stressors and severe relationship distress must be ruled out according to the DSM‐5 (see *D‐Criterion*, APA, 2013). Differences in the overall number of defined sexual dysfunctions, in the diagnostic threshold, as well as in the specification and operationalization of the disorder characteristics, but especially the diverging approach to the etiology of a sexual problem, can be expected to have a major impact on prevalence rates of sexual dysfunctions. More generally speaking, it will complicates the comparability of diagnostic decisions and research across classification systems (Schwesig, Briken, et al., 2022). To adequately address this issue, the DISEX takes the named conceptual differences into account and was developed to become applicable for the assignment of DSM‐5 as well as ICD‐11 diagnoses (see methods section for more detail).

### 1.4 The present study

In the present study, we aim to introduce the DISEX and its construction logic as well as report results of a first comprehensive validation study. Due to the abovementioned methodological and practical limitations of the existing measurements for sexual dysfunctions, common validation procedures comparing the results of a newly developed diagnostic tool to existing validated measures remain unsatisfactory. Another customary method is to use clinical‐psychiatric expert judgments as a validity criterion (see for example Osorio et al., 2019; Shabani et al., 2020). However, using expert judgment as a validity criterion for a diagnostic interview should be critically considered as unstandardized, clinical expert judgments are subject to validity and reliability deficiencies. Further, it was precisely this problem that was the starting point for the development of structured interviews (see In‐Albon et al., 2008). As an alternative, we developed a vignette‐based validation method involving standardized patients (actresses portraying pre‐defined diagnostic case vignettes; see DeRogatis et al., 2008, for a similar approach). Importantly, standardized patients provide unique advantages for instrument development: First, prototypical as well as rare patient representations/profiles can be easily included. Second, the prevalence of cases versus non‐cases as well as the prevalence of specific disorders can be controlled to maximize the informative value of the calculated psychometric criteria for each disorder. Furthermore, the patient‐side information can be hold almost completely constant over time so that standardized patients are also very well suited for assessing the inter‐rater reliability of a diagnostic instrument (see also Badger et al., 1995; Derogatis et al., 2008). Thus, standardized patients provide an excellent environment (“clinical laboratory”, Derogatis et al., 2008) for the starting point of a sequential, multi‐step validation process.

Due to feasibility considerations, the first validating study of the DISEX concentrates on the female version of the interview (DISEX‐F). Criterion validity (sensitivity and specificity) of the DISEX‐F is measured as the agreement between the diagnoses assigned by diagnosticians using the DISEX‐F and the target diagnoses (or non‐cases) pre‐determined in the case vignettes. Importantly, for each case vignette, a diagnosis according to ICD‐11 and DSM‐5 diagnostic criteria was pre‐determined. Next to criterion validity, we assess inter‐rater reliability and the interviewers' evaluation (“acceptance”) of the DISEX‐F itself. In case of mismatch between the pre‐determined target diagnoses and the assigned diagnoses, we aim to analyses the source of the error. To control for the external validity of our validation method, we assess the authenticity of the interview situation. In line with the COSMIN guidelines for evaluating measurement properties of health‐related outcome measures (e.g., Mokkink et al., 2010), we pre‐defined lower limits for the magnitude of psychometric criteria that the DISEX‐F should at least reach to be considered a clinical reliable and valid diagnostic instrument. Taken feasibility considerations for maximum sample size into account, in accordance with the consistent convention to classify kappa values from *k* ≥ 0.40 onwards as at least moderate agreement (see Landis & Koch, 1977), we chose k = 0.40 as the lowest acceptable threshold for inter‐rater reliability. Based on suggestions from Bujang and Adnan (2016) and feasibility considerations, we chose 65% as the lowest acceptable threshold for sensitivity and specificity.

## 2 METHODS

Before data collection, this study was preregistered (see https://doi.org/10.17605/OSF.IO/8XK9Q). Sample size (number of case vignettes as well as number of ratings per case vignette) was calculated following a multi‐steps procedure using “irr” package (version 0.84.1, Gamer et al., 2019) and “MKpower” package (version 0.5, Kohl, 2020) in R. Sample calculation included a compensation for data thighs resulting from multiple ratings of the same case vignette (see preregistration document for a detailed description on how sample size was determined).

### 2.1 Participants

#### 2.1.1 Diagnosticians

A total of *N =* 16 diagnosticians (*n*_female_ = 11; *n*_male_ = 5) were recruited for the study. Diagnosticians were recruited from the Institute for Clinical Psychology and Psychotherapy of the Technische Universität Dresden and from local training institutes for psychotherapy. *N* = 7 diagnosticians were master students in clinical psychology with prior systematic training and clinical experience in conducting structured clinical interviews for mental disorders, *n* = 9 diagnosticians were clinical psychologists holding a master's degree and currently either attending an advanced training in cognitive behavioral therapy (CBT) or being already fully licensed as a CBT psychotherapist (*n* = 1). All diagnosticians were required to complete a systematic and standardized three‐hours training: First, diagnosticians deepened their theoretical background knowledge on sexual dysfunctions in women and on their classification in the ICD‐11 and DSM‐5. Second, diagnosticians learned and practiced the correct implementation and evaluation of the DISEX‐F. For this purpose, role‐playing exercises were mandatory. Diagnosticians received no monetary honorarium for their participation.

#### 2.1.2 Standardized patients

A total of *N* = 32 lay actresses were recruited, for example, by contacting several (amateur) acting groups in the area and through online advertisements. Prerequisites for participation was an age over 18 and fluency in German language. Each case vignette was portrayed by an actress who roughly matched the age of the fictional patient. Case vignettes were sent to the actresses approximately one week before the study session. In preparation for their role, actresses received coaching and detailed information on sexual dysfunctions. Actresses were aged 19–72 years old and obtained monetary compensation for their study participation.

### 2.2 Material and measurements

#### 2.2.1 DISEX‐F for DSM‐5 and ICD‐11

The DISEX‐F (Schwesig, Velten, & Hoyer, 2022) is a freely accessible clinical interview for the assessment and categorical diagnosis of sexual dysfunctions applicable in clinical practice and research. Two separate evaluation sheets allow for the evaluation of the DISEX‐F according to either, DSM‐5 or ICD‐11. Additionally, the DISEX allows for the assessment of the subtype of the sexual dysfunction (generalized vs. situational; lifelong vs. acquired). The female version (DISEX‐F) captures all diagnostic criteria for the following disorder categories: ICD‐11: Hypoactive sexual desire dysfunction, Female sexual arousal dysfunction, Orgasmic dysfunctions, Sexual pain‐penetration disorder; DSM‐5: Female Sexual Interest/Arousal Disorder, Female Orgasmic Disorder, Genito‐Pelvic Pain/Penetration Disorder.

##### Development

The DISEX‐F reflects content and wording of the DSM‐5 and ICD‐11. During the development process, changes and additions to the DISEX were proposed, implemented, and tested by experts in the fields of sexual dysfunctions and several experienced clinical psychologists, who were all familiar with the DSM‐5 and the ICD‐11 criteria. While the DISEX‐F was developed and tested in a German research institute, its translation into English according to current guidelines is ongoing and its international availability will be provided.

##### Structure

The DISEX‐F is structured into three parts including questions on the following topics: 1. background information (e.g., demographic information, partnership and family status, psychosexual development); 2. current sexual problems (lack of/reduced sexual desire/arousal, orgasm difficulties, sexual pain/penetration difficulties) including frequency and severity of symptoms, symptom duration, and personal distress; and 3. etiology (causal or/and contributing factors to the described sexual problem).

#### 2.2.2 Case vignettes

The case vignettes comprised of 32 fictive patient descriptions in which criteria of predetermined target diagnoses were included a priori. The descriptions included a patient's demographic data, psychosocial background, symptoms, and contextual factors relevant to the described sexual problems (e.g., violence in the relationship or use of medication). Each case vignette was about 500 words long. Case vignettes were constructed so that each sexual disorder occurred at least twice as a single clinical diagnosis and was comorbid with one of the other sexual disorders at least eight times. Further, several case vignettes were constructed so that a sexual disorder should be assigned according to ICD‐11 criteria but should not be assigned according to DSM‐5 criteria (e.g., due to substance or medication use, a medical disease or severe relationship distress). The patient profiles were fictive but inspired by actual cases from local outpatient clinics and cases described in the DSM‐IV and DSM‐5 Case Books (Barnhill, 2014; Spitzer et al., 1994). Table 1 in the Supporting Information S1 lists the system of case vignettes that we used.

#### 2.2.3 DISEX‐adapted Interviewer Acceptance Questionnaire

To assess diagnosticians' acceptance of the DISEX‐F, we used the *Interviewer Acceptance Questionnaire* (IAQ; Suppiger et al., 2009). The questionnaire measures the acceptance of the diagnostic interview by 10 items using a 4‐point Likert scale ranging from 0 = *disagree* to 3 = *completely agree*. For adaption in this study, we modified the second item and split it into two separate items to be able to distinguish between the evaluation according to ICD‐11 and evaluation according to DSM‐5 (“I found it difficult to obtain the information needed for a DSM‐5 [ICD‐11] diagnosis with the help of the DISEX‐F.”).

#### 2.2.4 Authenticity of the interview situation

Three additional items were developed for this study to serve as feedback on the authenticity of the interview situation. In particular, the three items assess the authenticity of the standardized patient, the authenticity of the portrayed case, as well as the authenticity of the diagnostician‐patient‐situation. Answers are given on a 4‐point Likert scale ranging from 0 = *disagree* to 3 = *completely agree*.

### 2.3 Procedure

The standardized patients were assigned to the diagnosticians according to availability. Diagnosticians and standardized patients provided their written informed consent upon arrival at the interview location. Each standardized patient was interviewed independently and consecutively by two diagnosticians. After conducting the interview, diagnosticians evaluated the interview according to both, ICD‐11 and DSM‐5, by using the two separate evaluation sheets (Rating 1 and 2). The order in which they filled out the two evaluation sheets was not controlled for. The second interview and its evaluation were conducted blinded to the results of the first interview. Then, diagnosticians filled in the DISEX‐adapted IAQ and answered the three additional items measuring the authenticity of the situation. To expand the data base for inter‐rater reliability, both interviews were videotaped. One of the two videotapes was randomly selected for further evaluation: Two other diagnosticians watched the videotape and evaluated the interview independently (Rating 3 and 4). After each video evaluation, diagnosticians filled out a shortened version of the DISEX‐adapted IAQ and answered the three items on the authenticity of the situation. Interviews lasted on average *M* = 32.79 min (*SD* = 9.15 min, Range: 17–65 min). During the study, each diagnostician conducted four interviews (except for one diagnostician who conducted three and one who conducted five). Four of the 16 diagnosticians also took part in rating the videotapes.

### 2.4 Statistical analyses

Sensitivity, specificity, and inter‐rater reliability (Cohen's and Fleiss' kappa) were calculated separately for each sexual dysfunction in the ICD‐11 and DSM‐5. To communicate precision of the estimates and to test our hypotheses (sensitivity and specificity ≥ 0.65, Cohen's and Fleiss' kappa *k* ≥ 0.4), we calculated one‐sided confidence intervals (CIs). To obtain one‐sided 95% CIs, we calculated two‐sided 90% CIs, but only evaluated the lower limits while setting the upper limit to 1. Further, we analyzed the sources of mismatch between the diagnoses assigned by diagnosticians using the DISEX‐F and the pre‐determined target diagnoses included in the case vignettes. To that end, based on error categories identified in other validation studies (Neuschwander et al., 2013; Suppiger et al., 2008), a checklist with error categories was created post‐hoc but before error analysis. For each false diagnostic decision (that is, someone either failed to assign a diagnosis or incorrectly assigned one), the main source of error was identified. In addition, we assessed the authenticity of the interview situation and the acceptance of the DISEX‐F among diagnosticians by calculating mean and standard deviations as well as the distribution of given answers for each item. To explore whether ratings of difficulty of the diagnostic decision depended on the classification system (DSM‐5 vs. ICD‐11), a two‐tailed Wilcoxon signed rank test was conducted. Data of the study as well as the analysis code to reproduce all statistical results are freely accessible under osf.io/zcqwm.

## 3 RESULTS

### 3.1 Authenticity of the interview situation

Importantly, although standardized patients were used instead of actual patients, diagnosticians still rated the entire interview situation as highly authentic: Diagnosticians almost completely or completely agreed that the diagnostician‐patient situation seemed natural (94% of ratings), that the patient portrayed appeared authentic (98% of ratings), and that the case presented seemed realistic (97% of ratings). Full descriptive statistics of the ratings can be found in Table 2 in the Supporting Information S1.

### 3.2 Psychometric criteria

#### 3.2.1 Sensitivity and specificity

All specificity estimates were satisfactory (≥0.90) with all corresponding lower limits of CIs being in a clinically acceptable range (≥0.65). This was also the case for most sensitivity estimates. However, the estimates of the *Female Sexual Interest/Arousal Disorder* (DSM‐5), the *Female Orgasmic Disorder* (DSM‐5) as well as the *Female sexual arousal dysfunction* (ICD‐11) were clinically unsatisfyingly low (lower CI limits ≤ 0.65; see Table 1).

**TABLE 1:** Validity of the DSM‐5 and ICD‐11 diagnoses using the DISEX‐F.

	Frequencies		PPV (%)	NPV (%)	Sensitivity	Specificity
	−/−	−/+				
	+/−	+/+				
DSM‐5						
Female Sexual Interest/Arousal Disorder	61	36	77	63	0.40 [0.29, 1]	0.90 [0.82, 1]
	7	24				
Female Orgasmic Disorder	73	13	93	85	0.75 [0.63, 1]	0.96 [0.90, 1]
	3	39				
Genito‐Pelvic Pain/Penetration Disorder	79	4	98	95	0.92 [0.82, 1]	0.99 [0.94, 1]
	1	44				
ICD‐11						
Hypoactive sexual desire dysfunction	75	4	98	95	0.92 [0.83, 1]	0.99 [0.94, 1]
	1	48				
Female sexual arousal dysfunction	72	15	90	83	0.71 [0.59, 1]	0.95 [0.88, 1]
	4	37				
Orgasmic dysfunctions	59	15	98	80	0.78 [0.68, 1]	0.98 [0.92, 1]
	1	53				
Sexual pain‐penetration disorder	75	2	98	97	0.96 [0.88,1]	0.99 [0.94, 1]
	1	50				

*Note*: −/− = correct negative, −/+ false negative, +/− false positive, +/+ = correct positive. Brackets indicate one‐sided 95% CIs.

Abbreviations: NPV, negative predictive value; PPV, positive predictive value.

#### 3.2.2 Inter‐rater reliability

Inter‐rater reliability was calculated for the agreement between the two diagnosticians who each conducted an interview consecutively with the same standardized patient (“live”; Cohen's kappa) as well as for the agreement between the diagnostician conducting the interview and the two diagnosticians watching the videotaped interview (“video”; Fleiss' kappa). When the DISEX‐F was evaluated according to ICD‐11 criteria, all kappa estimates were excellent (*k* ≥ 0.75) and their corresponding CIs were in a clinical acceptable range (all lower limits ≥ 0.4). Kappa values of the DSM‐5 evaluation were mostly lower but still clinically acceptable (*k* ≥ 0.66 with all lower CI limits above 0.4) except for the *Female Sexual Interest/Arousal Disorder* (*k* = 0.44, lower CI limit ≤ 0.4 for both estimates, “live” and “video”; see Table 2).

**TABLE 2:** Inter‐rater reliability of the DSM‐5 and ICD‐11 diagnoses using the DISEX‐F.

	“Live”				“Video”
	Frequencies		%	Cohen's kappa	Fleiss' kappa
	−/−	−/+			
	+/−	+/+			
DSM‐5					
Female Sexual Interest/Arousal Disorder	20	4	78	0.44 [0.13; 1]	0.50 [0.33; 1]
	3	5			
Female Orgasmic Disorder	18	2	84	0.66 [0.43; 1]	0.76 [0.59; 1]
	3	9			
Genito‐Pelvic Pain/Penetration Disorder	20	0	100	1.00 [1; 1]	0.77 [0.60; 1]
	0	12			
ICD‐11					
Hypoactive sexual desire dysfunction	18	1	90	0.80 [0.63; 1]	0.96 [0.79; 1]
	2	11			
Female sexual arousal dysfunction	20	2	91	0.79 [0.60; 1]	1.00 [0.83; 1]
	1	9			
Orgasmic dysfunctions	16	1	88	0.75 [0.55; 1]	1.00 [0.83; 1]
	3	12			
Sexual pain‐penetration disorder	18	1	97	0.94 [0.83; 1]	0.91 [0.74; 1]
	0	13			

*Note*: −/− = correct negative, −/+ false negative, +/− false positive, +/+ = correct positive; % = percentage of agreement; “Live” = agreement between the two diagnosticians who each conducted an interview consecutively with the same standardized patient. “Video” = agreement between the diagnostician conducting the interview and the two diagnosticians watching the videotaped interview. Brackets indicate one‐sided 95% CIs.

### 3.3 Source of mismatch between the pre‐determined target diagnoses and the assigned diagnoses

In total, 8.4% of ICD‐11 evaluations, that is 43 out of 512 (32 case vignettes * 4 rater * 4 disorders) diagnostic decisions were false. For DSM‐5 evaluation, 16.7%, that is 64 out of 384 (32 case vignettes * 4 rater * 3 disorders) diagnostic decisions were false. Overall, diagnosticians made only few interview, logging, or evaluation errors. However, specific to DSM‐5 evaluation, the need to judge the etiology of a sexual problem led to relatively many false diagnostic decisions, especially with respect to differential diagnosis. Errors that were not related to the diagnosticians or the DISEX‐F itself but were study design specific, especially errors which occurred due to imprecise acting, accounted for 36.0% (DSM‐5) and 74.4% (ICD‐11) of false diagnostic decisions (see Table 3).

**TABLE 3:** Source of mismatch between the pre‐determined target diagnoses and the assigned diagnoses.

Type of error	DSM‐5					ICD‐11					
	IA^a^	O^d^	P^e^	In total	%	I^b^	A^c^	O^d^	P^e^	In total	%
Interview error (in total)	0	1	0	1	1.6	1	1	4	0	6	14.0
Jump rule incorrectly applied	0	0	0	0	0	1	1	0	0	2	4.7
Strong deviation from standardized questions	0	0	0	0	0	0	0	0	0	0	0
Insufficient follow‐up questions asked	0	0	0	0	0	0	0	0	0	0	0
Other reasons	0	1	0	1	1.6	0	0	4	0	4	9.3
Logging error (in total)	0	1	2	3	4.7	0	1	1	2	4	9.3
Interview guide	0	1	2	3	4.7	0	0	1	2	3	7.0
Evaluation sheet	0	0	0	0	0	0	1	0	0	1	2.3
Evaluation error (in total)	16	10	3	29	45.3	0	0	0	1	1	2.3
K or M‐criteria applied wrongly^f^	1	0	0	1	1.6	0	0	0	0	0	0
Incorrect differential diagnostic decision	15	10	3	28	43.8	0	0	0	0	0	0
Incorrect final classification despite correct exclusion	0	0	0	0	0	0	0	0	1	1	2.3
Imprecision in case vignette (Unclear/ambiguous/missing information; in total)^g^	8	0	0	8	12.5	0	0	0	0	0	0
Imprecise acting (in total)^h^	19	4	0	23	36.0	4	17	11	0	32	74.4
Incorrect information regarding symptom severity	10	0	0	10	15.6	0	14	7	0	21	48.8
Incorrect information regarding symptom burden	9	0	0	9	14.1	4	3	0	0	7	16.3
Omission of relevant information	0	0	0	0	0	0	0	0	0	0	0
Adding of case modifying information	0	4	0	4	6.3	0	0	4	0	4	9.3
Quality of sound and video recording (in total)	0	0	0	0	0	0	0	0	0	0	0
In total	43	16	5	64^i^	100	5	19	16	3	43^j^	100

*Note*: Note, that especially for interview errors, imprecise acting, and imprecisions in case vignettes, one error often inevitably influenced all three or four ratings.

^a^ IA = Female Sexual Interest/Arousal Disorder.

^b^ I = Hypoactive sexual desire dysfunction.

^c^ A = Female sexual arousal dysfunction.

^d^ O = Female Orgasmic Disorder (DSM‐5) or Orgasmic dysfunctions (ICD‐11).

^e^ P = Genito‐Pelvic Pain/Penetration Disorder (DSM‐5) or Sexual pain‐penetration disorder (ICD‐11).

^f^ K‐criteria = symptom criterion including personal suffering, M‐criteria = inclusion criteria (e.g., adequate stimulation).

^g^ This type of error was found in 2 out of 32 case vignettes.

^h^ This type of error was found for 10 out of 32 actresses.

^i^ there were *n* = 384 (DSM‐5; 32 case vignettes * 4 rater * 3 disorders) and *n* = 512 (ICD‐11; 32 case vignettes * 4 rater * 4 disorders) possibilities for the occurrence of errors.

### 3.4 Interviewer acceptance of the DISEX‐F

The responses from the DISEX‐adapted *IAQ* indicate generally high acceptance among interviewers: 88.9% of diagnosticians perceived the relationship with the patient as pleasant (completely or almost completely agreed), 80.7% indicated that they could respond adequately to the patient during the interview, and less than 1.6% of interviews were described as exhausting (see Table 4). To explore whether ratings on the difficulty of the diagnostic decision depended on the classification system (DSM‐5 vs. ICD‐11), a two‐tailed Wilcoxon signed rank test was conducted for which ratings of all diagnosticians (including the ratings from the videotapes; see Table 3 in the Supporting Information S1) were included. Results indicate a significant difference with the DSM‐5 evaluation (*M* = 0.52, *SD* = 0.71, *Md* = 0, *n* = 128) rated to be more difficult than the ICD‐11 evaluation (*M* = 0.27, *SD* = 0.54, *Md* = 0, *n* = 128), *V* = 22, *p* < 0.001, *r* = 0.38.

**TABLE 4:** Mean, standard deviation and distribution of answers of the DISEX‐adapted Interview Acceptance Questionnaire (Suppiger et al., 2009) rated by the diagnosticians directly after evaluating the conducted interviews (*N* = 64).

Item	*M* (*SD*)	Distribution of given answers			
		0	1	2	3
Positively formulated items					
I have conducted the interview to the best of my knowledge and ability.	2.80 (0.51)	0%	4.7%	10.9%	84.4%
While conducting the interview I felt competent.	2.06 (0.62)	0%	15.9%	61.9%	22.2%
The patient perceives herself and her problems in a differentiated manner.	2.39 (0.61)	0%	6.3%	48.4%	45.3%
I experienced the relationship with the patient as pleasant.	2.35 (0.68)	0%	11.1%	42.9%	46.0%
I succeeded in being responsive to the patient.	2.08 (0.76)	3.1%	15.6%	51.6%	29.7%
During the interview I experienced the patient as cooperative.	2.66 (0.54)	0%	28.1%	68.8%	0%
Negatively formulated items					
I made mistakes in administering the interview.	0.88 (0.79)	32.8%	51.6%	10.9%	4.7%
I found it difficult to obtain all information needed to assign a diagnosis according to ICD‐11 using the DISEX.	0.31 (0.53)	71.9%	25.0%	3.1%	0%
I found it difficult to obtain all information needed to assign a diagnosis according to DSM‐5 using the DISEX.	0.52 (0.64)	56.2%	35.9%	7.8%	0%
I found the interview too exhausting.	0.30 (0.49)	71.9%	26.6%	1.6%	0%
I think the patient did not report everything that was bothering her.	0.72 (1.00)	57.8%	21.9%	10.9%	9.4%

*Note*: Items were rated on a 4‐point scale ranging from 0 to 3 (0 = disagree, 1 = slightly agree, 2 = almost completely agree, 3 = completely agree).

## 4 DISCUSSION

The aim of the present study was to determine first results on the validity and reliability of the *Diagnostic Interview for Sexual Dysfunctions* *in Women* (DISEX‐F), which covers diagnostic criteria of DSM‐5 and ICD‐11. We used standardized patients to ensure the comprehensive assessment of sensitivity, specificity, and inter‐rater reliability of the DISEX‐F across all diagnostic categories.

Sensitivity of the DISEX‐F greatly depended on the classification system used for evaluation and the type of sexual dysfunction. Estimated sensitivity was mostly higher for ICD‐11 diagnoses than for DSM‐5 diagnoses. For most sexual dysfunctions, results indicate a high potential of the DISEX‐F to validly identify true positives (at least 78% probability). Yet, sensitivity estimates of the *Female Sexual Interest/Arousal Disorder* (DSM‐5), the *Female Orgasmic Disorder* (DSM‐5) as well as the *Female sexual arousal dysfunction* (ICD‐11) remained clinically unsatisfactory (lower CI limits ≤65%).

Specificity, that is the probability of the DISEX‐F to identify true negatives was found to be excellent (at least 90%) for all DSM‐5 and ICD‐11 disorders. Therefore, results fully support the potential of the DISEX‐F to minimize rates of false negative DSM‐5 and ICD‐11 diagnoses, an important strength as previous studies have shown that sexual dysfunction often remains underdiagnosed in primary health care services (e.g., Heiden‐Rootes et al., 2017; Ribeiro et al., 2014) and outpatient clinics (e.g., Hoyer et al., 2009; Velten et al., 2021).

Inter‐rater reliability of the DISEX‐F was substantial to almost perfect for all DSM‐5 and ICD‐11 diagnoses (*k* ≥ 66; see Landis & Koch, 1977) except for the *Female Sexual Interest/Arousal Disorder* (DSM‐5) for which the agreement was only moderate (*k* = 0.44; lower CI limit beneath 0.4).

Closer inspection of the mismatches between assigned diagnoses and pre‐determined diagnoses indicated that many mismatches were not attributable to the diagnosticians or the DISEX‐F itself but rather to imprecise acting of the actresses impersonating the patients. Specifically, imprecise acting partly explained the found low sensitivity estimates: For the *Female Sexual Interest/Arousal Disorder* (DSM‐5) 44% of mismatches, for the *Female Orgasmic Disorder* (DSM‐5) 25% of mismatches, for the *Female sexual arousal dysfunction* (ICD‐11) 89% of mismatches, and for *Orgasmic dysfunctions* (ICD‐11) 69% of mismatches were attributable to imprecise acting.

Importantly, diagnosticians made very few interview errors which shows that the correct conduction of the DISEX‐F can easily be learned in a relatively short training time. However, mixed results were found for the evaluation of the DISEX‐F: While the evaluation according to ICD‐11 criteria was mostly carried out perfectly, the DSM‐5 specific demand to judge the etiology of a sexual problem explained many of the false differential diagnostic decisions. The latter was especially a major source of error for those DSM‐5 diagnoses for which sensitivity and inter‐rater reliability estimates were found to be clinically unsatisfying: For the *Female Sexual Interest/Arousal Disorder* 35% of mismatch and for the *Female Orgasmic Disorder* even 63% of mismatch were attributable to false differential diagnostic decisions. The following two possible explanations for the frequent occurrence of a false differential diagnostic decision according to DSM‐5 should be considered. First, the DSM‐5 *D‐Criterion* is imprecisely operationalized in the APA manual: It remains largely unclear what is meant exactly by “severe relationship distress” and “significant stressors” (APA, 2013). As the DISEX‐F is meant to directly reflect wording and content of the DSM‐5, there is no additional specification of the *D‐Criterion* in the DISEX‐F. Consequently, this might have led to diverging clinical decisions on whether the *D‐Criterion* was met or not. In line with this explanation, authors of other validation studies of structured interviews reported low sensitivity, especially when DSM‐5 diagnostic criteria were unsatisfactorily operationalized (In‐Albon et al., 2008; Suppiger et al., 2008). Second, as the DSM‐5 requires a clinical judgment on whether certain background factors are irrelevant, have caused or maintained the sexual problem, diagnosing sexual dysfunctions according to DSM‐5 demands clinical expertise in the field of sexual dysfunctions. Therefore, false differential diagnostic decisions should not necessarily be seen as a problem of the DISEX‐F per se. Instead, they are attributable to unsatisfactory operationalization of the *D‐Criterion* in the DSM‐5 and/or high demands of expertise required by the DSM‐5 classification system. Clinical expertise in the fields of sexual dysfunctions as well as a further specification of the wording of the *D‐Criterion* in the DSM‐5 would surely enhance validity and reliability of DSM‐5 diagnoses. In addition, a present medical consultation report on the influence of medical factors (e.g., diseases, drugs) on the reported symptoms could further enhance the correct evaluation of etiological factors and strengthen the diagnostician's certainty in the differential diagnostic decision. Taken together, while evaluating the DISEX‐F according to ICD‐11 can be validly done by trained but non‐specialized clinical staff, the evaluation according to DSM‐5 is more prone to error and demands a higher level of knowledge and experience.

Diagnosticians' evaluation of the DISEX‐F was highly positive: Acceptance ratings of the DISEX‐F for diagnostic purposes were found to be generally high among all diagnosticians. Our result corroborates findings of previous studies that showed that structured interviews are well accepted among diagnosticians (e.g., Neuschwander et al., 2017; Suppiger et al., 2009). Interestingly, as explanatory analysis indicated, evaluation of the DISEX‐F according to DSM‐5 criteria was perceived to be more difficult than evaluating it according to ICD‐11 criteria. This might reflect the uncertainties caused by the *D‐Criterion* of the DSM‐5. Whether the acceptance ratings are generalizable to clinical experts in inpatient, outpatient, and research settings, will still need to be investigated.

### 4.1 Limitations and further research suggestions

The following limitations of our study need to be considered: First, most diagnosticians had limited clinical experience in diagnosing and treating sexual dysfunctions. While our results should be a good indication of the psychometric properties of the DISEX‐F administered by trained but non‐specialized clinical staff, our result might underestimate the reliability and validity estimates of the DISEX‐F when administered by experts in sexual dysfunctions. As all diagnosticians in the present study underwent a training, the results cannot be generalized to untrained clinicians.

Second, although acceptance ratings and authenticity ratings were given anonymously, they might be influenced by social desirability. The latter, however, is a common limitation of studies investigating opinions of diagnosticians and our study results should therefore be comparable to similar research (e.g., Neuschwander et al., 2017; Suppiger et al., 2009).

Third, due to methodological considerations, the DISEX‐F was tested using standardized patients. In addition to establishing pre‐determined diagnoses, this enabled us to control and manipulate patients' profiles, for example, the (co‐)occurrence of symptoms, symptom duration, personal suffering, as well as causal and maintaining factors. An important strength of the study design is the inclusion of a heterogeneous sample of case profiles. However, although the interview situation was perceived as highly authentic, compared to a standard diagnostic situation, the information provided by standardized patients might be more explicit and definite and less depending on factors of the patient‐interviewer‐relationship and the interview situation (e.g., shame, information bias due to social desirability, see also DeRogatis et al., 2008). Furthermore, in the case vignettes, information on causal and maintaining factors were explicitly given (e.g., results from medical examinations). This might also be otherwise less definitive information given by real patients. Unfortunately, almost one third of actresses deviated, at least slightly, from the descriptions in the case vignettes and gave incorrect information regarding symptom severity and symptom burden. This could, however, potentially reflect natural variability in reporting, which generally limits the realistic expectancy value of test indices (see also Suppiger et al., 2008: 42% of mismatch for test‐retest‐reliability due to patient information variance).

Overall, the use of standardized patients and mainly fictitious profiles (instead of actual patients) might have led to a bias in the estimation of reliability and validity and, as a result, to limited ecological validity. On the other hand, factors that limit the interpretability of validation studies in routine practice, such as the oversampling of certain diagnoses, while other diagnoses are not represented, could be well controlled in our study. While first validating steps were made with this study, it is crucial to continue the validation process including affected women and to assess its clinical validity in routine care. To this end, the following research suggestions should be considered: (1) to assess convergent and discriminant validity of the DISEX‐F via correlations with psychometric measures such as the FSFI (Rosen et al., 2000) and the Sexual Interest and Desire Inventory‐Female (SIDI‐F; Clayton et al., 2006), (2) to examine test‐retest reliability of the DISEX‐F, and (3) to evaluate acceptance of the DISEX‐F among actual patients/women and among experienced licensed psychotherapists and sexual medical experts.

## 5 CONCLUSION

The DISEX‐F was constructed to fill the gap of a practical and valid diagnostic instrument for sexual dysfunctions in clinical practice and research. Overall, the present results confirm that the DISEX‐F is an easy acquirable and promising tool to enhance identification of sexual dysfunctions in women: Despite the short training of the diagnosticians, we found high specificity for all ICD‐11 and DSM‐5 disorders, and high sensitivity and inter‐rater reliability for most ICD‐11 disorders. Mixed results were found for sensitivity and inter‐rater reliability of DSM‐5 disorders. Validity and reliability of the DISEX‐F for DSM‐5 defined disorders might be restricted as long as the exclusion criteria defined in DSM‐5 remain relatively imprecise and are, hence, more prone to error. Further investigation of psychometric properties of the DISEX‐F involving patient samples is clearly indicated as only the existence of a valid and reliable diagnostic instrument for sexual dysfunctions can enable comparability, transferability, and replicability of clinical diagnoses, and, therefore, be a reliable foundation of productive research and effective treatments.

## AUTHOR CONTRIBUTIONS

**Rebekka Schwesig**: Conceptualization; data curation; formal analysis; funding acquisition; investigation; methodology; project administration; resources; supervision; validation; visualization; writing—original draft; writing—review and editing. **Julia Velten**: Validation; writing—original draft; writing—review and editing. **Jurgen Hoyer**: Conceptualization; methodology; project administration; resources; supervision; writing—original draft; writing—review and editing.

## CONFLICT OF INTEREST STATEMENT

The authors report no conflicts of interest.

## ETHICS STATEMENT

The study received ethical approval from the ethics committee of the Technische Universität Dresden (SR‐EK‐139032022). All participants (actresses and diagnosticians) provided written informed consent before participation.

## Supporting information

Supporting Information S1

## Data Availability

The data that support the findings of this study are openly available via the Open Science Framework at osf.io/zcqwm
