# Supplementary material for: The Diagnostic Interview for Sexual Dysfunctions in Women for DSM‐5 and ICD‐11: Development and initial validation using a vignette‐based approach
Source: Int J Methods Psychiatr Res. 2023 Dec 28;33(1):e2004. doi: 10.1002/mpr.2004 (PMC10754473; doi:10.1002/mpr.2004)
Supplement: Supplementary file 1 — Supporting Information S1 [file MPR-33-e2004-s001.docx]

**Supplementary Material**

**Table 1**

*List of Case Vignettes and Included Diagnoses*

| **Case vignette** | **DSM-5** | | | **ICD-11** | | | |
| --- | --- | --- | --- | --- | --- | --- | --- |
|  | **IA^1^** | **O^4^** | **P^5^** | **I^2^** | **A^3^** | **O^4^** | **P^5^** |
| 1 | X |  |  | X |  |  |  |
| 2 |  |  |  | X |  |  |  |
| 3 | X |  |  | X |  |  |  |
| 4 | X |  |  | X |  |  |  |
| 5 | X |  |  |  | X |  |  |
| 6 |  |  |  |  | X |  |  |
| 7 |  | X |  |  |  | X |  |
| 8 |  | X |  |  |  | X |  |
| 9 |  | X |  |  |  | X |  |
| 10 |  | X |  |  |  | X |  |
| 11 |  |  | X |  |  |  | X |
| 12 |  |  | X |  |  |  | X |
| 13 |  |  | X |  |  |  | X |
| 14 |  |  | X |  |  |  | X |
| 15 |  | X |  | X | X | X |  |
| 16 |  |  |  | X |  | X |  |
| 17 |  |  |  | X | X | X |  |
| 18 | X | X |  | X | X | X |  |
| 19 | X | X |  | X | X | X |  |
| 20 | X |  | X | X |  |  | X |
| 21 | X |  | X | X |  |  | X |
| 22 | X |  | X | X |  |  | X |
| 23 | X | X |  |  | X | X |  |
| 24 |  |  |  |  | X | X |  |
| 25 | X | X |  |  | X | X |  |
| 26 | X | X |  |  | X | X |  |
| 27 | X |  | X |  | X |  | X |
| 28 |  |  | X |  | X |  | X |
| 29 | X | X | X |  | X | X | X |
| 30 |  | X | X |  |  | X | X |
| 31 |  |  |  |  |  | X | X |
| 32 | X | X | X | X |  | X | X |
| Incidence | 15 | 13 | 12 | 13 | 13 | 17 | 13 |
| Prevalence | 46.9% | 40.6% | 37.5% | 40.6% | 40.6% | 53.1% | 40.6% |

*Note*. ^1^IA= *Female Sexual Interest/Arousal Disorder*; ^2^I = *Hypoactive sexual desire dysfunction*; ^3^A= *Female sexual arousal dysfunction*; ^4^O= *Female Orgasmic Disorder* (DSM-5) or *Orgasmic dysfunctions* (ICD-11), ^5^P= *Genito-Pelvic Pain/Penetration Disorder* (DSM-5) or *Sexual pain-penetration disorder* (ICD-11). Cases in which ICD but not DSM diagnoses are present mainly appear when the DSM-5 *D-Criterion* is not fulfilled.

**Table 2**

*Authenticity of the Interview Situation****:*** *Mean, Standard Deviation, and Distribution of Answers (N=64)*

|  |  | **Distribution of given answers** | | | |
| --- | --- | --- | --- | --- | --- |
| **Item** | ***M (SD)*** | **0** | **1** | **2** | **3** |
| The diagnostician-patient situation seemed unnatural to me. | 0.28  (0.61) | 78.7% | 15.7% | 3.9% | 1.6% |
| ﻿The patient portrayed appeared authentic to me. | 2.74 (0.47) | 0% | 1.6% | 22.7% | 75.8% |
| The case presented seemed realistic to me. | 2.82 (0.49) | 0.8% | 2.3% | 10.9% | 85.9% |

*Note:* ﻿Items were rated on a 4-point scale ranging from 0 to 3 (0 = *disagree*, 1 = *slightly agree*, 2 = *almost completely agree*, 3 = *completely agree*).

**Table 3**

*Mean, Standard Deviation and Distribution of Answers of the DISEX-adapted* Interview Acceptance Questionnaire *(Suppiger et al., 2009) Rated by the Diagnosticians Directly After Evaluating the Videotaped Interviews (N= 64)*

|  |  | **Answer** | | | |
| --- | --- | --- | --- | --- | --- |
| **Item** | ***M***  ***(SD)*** | **0** | **1** | **2** | **3** |
| **Positively formulated items** | | | | | |
| ﻿The patient perceives herself and her problems in a differentiated manner. | 2.70 (0.55) | 0 % | 4.69 % | 20.31% | 75.00% |
| ﻿During the interview I experienced the patient as cooperative. | 2.83 (0.52) | 1.56% | 1.56% | 9.38% | 0% |
| **Negatively formulated items** | | | | | |
| ﻿﻿I found it difficult to obtain all information needed to assign a diagnosis according to ICD-11 using the DISEX | 0.22 (0.55) | 82.81% | 14.06% | 1.56% | 1.56% |
| I found it difficult to obtain all information needed to assign a diagnosis according to DSM-5 using the DISEX | 0.52 (0.78) | 62.50% | 26.56% | 7.813% | 3.13% |
| ﻿I think the patient did not report everything that was bothering her | 0.34 (0.86) | 82.81% | 7.81% | 1.56% | 7.81% |

*Note:* ﻿ Items were rated on a 4-point scale ranging from 0 to 3 (0 = *disagree*, 1 = *slightly agree*, 2 = *almost completely agree*, 3 = *completely agree*).
